# Association of vascular endothelial growth factor, transforming growth factor beta, and interferon gamma gene polymorphisms with proliferative diabetic retinopathy in patients with type 2 diabetes

**Published:** 2012-11-17

**Authors:** Suman Kalyan Paine, Analabha Basu, Lakshmi Kanta Mondal, Aditi Sen, Subhadip Choudhuri, Imran Hussain Chowdhury, Avijit Saha, Gautam Bhadhuri, Ankur Mukherjee, Basudev Bhattacharya

**Affiliations:** 1 Department of Biochemistry, Dr. B. C. Roy Post Graduate Institute of Basic Medical Education and Research (IPGME&R), Kolkata, India; 2 National Institute of Bio Medical Genomics, Kalyani, Nadia; 3 Regional Institute of Ophthalmology, Kolkata, India

## Abstract

**Purpose:**

Chronic hyperglycemia and hypoxemia are believed to be causal factors in the development of proliferative diabetic retinopathy (PDR) among individuals with type 2 diabetes. It is hypothesized that formation of new blood vessels in the retina due to prolonged hypoxia is associated with increased expression of several growth factors and angiogenic cytokines. In the present study, we investigated the association of genetic polymorphisms in vascular endothelial growth factor (VEGF), transforming growth factor beta (TGF-β), and interferon γ (IFN-γ) genes, which may be responsible for the hypoxia-induced VEGF-mediated neovascularization pathway for the pathogenesis of PDR.

**Methods:**

Our case-control association study composed of 493 ethnically matched volunteers (253 with PDR [cases] and 240 diabetic controls [DC]). Gene polymorphisms were determined with Taqman-based real-time PCR and amplification refractory mutation analysis system PCR.

**Results:**

The VEGF-460C (rs833061C; p=0.0043) and IFN-γ +874T (rs2430561T; p=0.0011) alleles were significantly associated with PDR.

**Conclusions:**

Genetic variations at VEGF-460C and IFN-γ +874T might accelerate the pathogenesis of retinal neovascularization in PDR.

## Introduction

Diabetic retinopathy (DR) is the most common microvascular complication of type 1 and type 2 diabetes mellitus (DM) and the most frequent single cause of new cases of blindness among adults in the 20- to 75-year age group [1]. DM is estimated to affect 4% of the world population, and retinopathy occurs in almost all patients with type 1 DM and 75% of patients with type 2 DM within 15 years of the manifestation of diabetes [2,3]. Visual loss develops primarily from either increased permeability of retinal vessels (diabetic macular edema) or proliferation of new retinal vessels.

Chronic hyperglycemia and hypoxemia are the two most important contributors to the development of proliferative diabetic retinopathy (PDR), leading to increased vasopermeability, endothelial cell proliferation, and undesired pathological neovascularization [4,5]. New blood vessel formation in the retina due to prolonged hypoxia is believed to be directly associated with increased expression of several pathoangiogenic growth factors such as vascular endothelial growth factor (VEGF), platelet derived growth factor (PDGF), transforming growth factor beta (TGF-β), and basic fibroblast growth factor (bFGF). Among these various growth factors, VEGF is considered the most potent angiogenic mediator in the genesis of several diseases, including retinal neovascularization in patients with type 2 diabetes [6]. TGF-β is another multifunctional growth factor that has an important role in modulating cell behavior in ocular tissues.

TGF-β has a role in modulating cell migration, proliferation, and protein synthesis during several physiologic and pathological processes [7]. TGF-β also acts as a chemoattractant for various cell types and is capable of producing several angiogenic factors such as VEGF, PDGF, and tumor necrosis factor (TNF-α), which accelerate the neovascularization process in the prolonged hyperglycemic condition [2,8]. Proinflammatory cytokines such as TNF-α and interferon γ (IFN-γ) generated by phagocytic cells upon cellular activation are also known to be angiogenic, fibrogenic, and vasculoreactive [8]. TNF-α may mediate alteration of vasoregulation and leukocyte adhesion, resulting in endothelial dysfunction and increased endothelial permeability. TNF-α may also play an important role in cell invasion and migration during angiogenesis [2,8]. IFN-γ is expressed at high levels in ocular tissues among patients with PDR and is considered an indirect inducer of angiogenesis through the activation of VEGF [8].

Since PDR is a microvascular complication associated with long-term complications of type 2 (and type 1) diabetes, the disease etiology is considered multigenic and complex with genetic and environmental factors. Thus, studying the effect of genetic alterations on the hypoxia-induced VEGF-mediated neovascularization pathway is imperative to better understand the pathophysiology of PDR. Moreover, since the involvement of cytokines in PDR is hypothesized, we took a candidate gene approach in designing a case-control association study of single nucleotide polymorphisms (SNPs) in IFN-γ, TGF-β1, and VEGF genes.

## Methods

This case-control study included 253 patients with PDR as a long-term complication of type 2 diabetes mellitus and 240 age, sex, nutrition, and glycemic level matched type 2 diabetic controls (duration of DM 17±5 years) without retinopathy. Patients with PDR were recruited at the retina clinic at the Regional Institute of Ophthalmology, Kolkata, India, and the controls were recruited at the diabetic clinic at the Institute of Post-graduate Medical Education and Research, Kolkata, India. All the study patients belonged to the same community (same ethnic group), Bengali Hindu, living in geographical proximity and are assumed to be pan-mixing. Institutional ethical clearance and written informed consent from each subject were obtained according to the Declaration of Helsinki.

Diagnosis of DM was made according to World Health Organization criteria [9]. PDR was diagnosed with dilated fundus examination with slit-lamp biomicroscopy by +90 D and three mirror lens and seven field digital fundus photography with fluorescence angiography. Grading or scale of severity of retinopathy was based on a modified version of the Early Treatment Diabetic Retinopathy Study (ETDRS).

People with coronary artery disease (CAD), hypertension, peripheral vascular diseases, history of any thrombotic event, acute infection, or any other ocular disorder such as glaucoma, branch retinal venous occlusion, or Eales disease were excluded from the study. To exclude patients with diabetic nephropathy, patients with microalbumin-creatinine ratio >30 mg/gm and urinary microalbumin level >300 mg /day were excluded from the study [10]. Venous blood samples were collected by venipuncture from the study patients, and genomic DNA was isolated from peripheral blood mononuclear cells (PBMC) with the conventional phenol-chloroform method [11].

### Genotyping with Taqman-based real-time polymerase chain reaction

Genotyping for VEGF-460T/C (rs833061) and TGF-β1–509C/T (rs1800469) polymorphisms was performed using a predesigned Taqman-based real-time PCR assay kit (Applied Biosystems, Foster City, CA; Catalog no. C1647381_10 and C8708473_10 respectively).

### Genotyping with amplification refractory mutation system-polymerase chain reaction

IFN-γ +874A/T (rs2430561) genotype analysis was conducted with amplification refractory mutation system-polymerase chain reaction (ARMS-PCR) [12,13]. Primers used for the amplification were 5′-TCA ACA AAG CTG ATA CTC CA-3′ (common reverse), 5′-TTC TTA CAA CAC AAA ATC AAA AAT CA-3′ (IFN-γ +874A allele specific), and 5′-TTC TTA CAA CAC AAA ATC AAA TCT-3′ (IFN-γ +874T allele specific). The 796 bp amplicon (fragment from the third intron of the *HLA-DRB1* gene) was used as an internal control, and 5′-TGC CAA GTG GAG CAC CCA A-3′ and 5′-GCA TCT TGC TCT GTG CAG AT-3′ were used as forward and reverse primers, respectively.

Cycling conditions for IFN-γ +874A/T (rs2430561 A/T) were as follows: 95 °C for 10 min, followed by 15 cycles of 95 °C for 1 min, 65.5 °C for 1 min, 72 °C for 1 min, and then 25 cycles of 95 °C for 1 min, 60 °C for 1 min, 72 °C for 1 min, followed by a final 10 min at 72 °C.

All PCRs were done in 10 µl reaction volumes, and final reagent concentrations were as follows: 1X Dream Taq reaction buffer with 2 mM MgCl_2_ (Fermentas, Glen Burnie, MD), 250 µM each deoxyribonucleotide triphosphate, 1 µM each specific/common primer, 0.5 µM each control primer, and 0.25 units Dream Taq DNA Polymerase (Fermentas), and 20–100 ng DNA. PCR was performed using a Biometra TPersonal 48 Thermal Cycler (Goettingen, Germany). The amplified products were separated by electrophoresis on 2% agarose gel stained with 0.5 mg/ml ethidium bromide and visualized and photographed under an ultraviolet transilluminator.

### Statistical analysis

Age, blood pressure, urinary microalbumin level, microalbumin-creatinine ratio, glycemic, and nutritional status were compared between the study groups (PDR and DM) using the two-tailed Student *t* test. The genotype phenotype association was done using a χ^2^ test. The odds ratio (OR) and the 95% confidence interval (95% CI) were calculated, and p values were evaluated using Fisher’s exact test. The level of statistical significance was set at p<0.05, except tests in which the Bonferroni adjustment was applied. All the SNPs were analyzed to check if they were in Hardy–Weinberg equilibrium (HWE) using Haploview (version 3.32, 2005) [14].

Analysis was performed to investigate whether the two SNPs (rs833061 and rs2430561) have a joint significant effect on disease status. First, using logistic regression, the main effect of the SNPs was regressed out, and then using the residuals, a linear regression analysis was performed. For logistic regression, the dependent variable, i.e., case (PDR) and control (DC), were encoded as 1 and 0, respectively. Duration of diabetes and the SNPs were considered independent variables. The analysis was done assuming a dominance model. The high expressive genotype, i.e., a homozygous or heterozygous carrier of the rs833061 C and rs2430561 T alleles, as coded as 2, and the low expressive genotype, i.e., rs833061 TT and rs2430561 AA alleles, was coded as 1.

To investigate the joint effect of the three SNPs, combined genotype analysis was performed using logistic regression. Based on previous studies, we assumed a dominant model of inheritance for all three SNPs in the study. To carry out genotype combination analysis of the three loci, we defined a categorical variable (combination) with four categories according to the number of dominant genotypes present in the combination genotype of the three SNPs (3 if all three SNPs have a dominant genotype, 2 if two SNPs have dominant genotypes; 1 if only one of the SNPs has a dominant genotype, and 0 if none of the SNPs has a dominant genotype). Using this variable (combination), we fitted a logistic regression model by taking the variable as the predictor and regressing on the case control status. All statistical analyses were performed using the R package.

## Results

Demographic and clinical characterization of the study patients is presented in Table 1. There was no significant difference in age, sex, blood pressure, glycemic, and nutritional status between the two study groups.

**Table 1:** Clinical characteristics of the study groups.

Parameters	PDR (n=253)	DC (n=240)	P value
Age	52±15.0	54±12.0	0.1
Sex			
Male	133 (52.56%)	128(53.33%)	0.92
Female	120 (47.44%)	112 (46.67%)	
HBA_I_C (%)	7.5±1.2	7.6±1.3	0.37
Urinary micro albumin (mg/day)	19.02±5.94	18.1±6.08	0.09
Urinary micro albumin creatinine ratio (mg/gm)	18.45±4.96	17.54±5.64	0.06
Serum total protein (gm/dl)	7.24±1.02	7.1±0.88	0.1
Duration of diabetes (years)	15±8	17±5	0.001*
Blood pressure (mmHg)			
Systolic	130±9	131±8	0.08
Diastolic	82±6	82±5	0.054

* Significant at p<0.001 level, HBA_1_C means glycosylated hemoglobin.

The genotyping was done for the entire group of 253 patients with PDR and 240 controls (DC). No significant departure from HWE was observed at the rs833061 T/C, rs1800469 C/T, and rs2430561 A/T loci among the patients with PDR (χ^2^=3.654, p=0.0559; χ^2^=1.2414, p=0.2651; χ^2^=0.3187, p=0.5723; respectively) or the controls (χ^2^=0.0553, p=0.814; χ^2^=1.7183, p=0.1899; χ^2^=0.7271, p=0.3938; respectively). The genotypic and allelic distributions of rs833061 T/C, rs1800469 C/T, and rs2430561 A/T are given in Table 2.

**Table 2:** Genotypic/Allelic Difference between cases and controls.

dBSNP	Genotype	PDR n=253(%)	DC n=240(%)	OR(95%CI)	P
rs833061	TT	152(60.07)	167(69.58)	Ref	
	CT	81(32.01)	67(27.91)	1.32 (0.88–2.003)	0.16
	CC	20(7.92)	6(2.5)	3.66(1.35–11.4)	0.004*
	T	385(76.08)	401(83.54)	Ref	
	C	121(23.92)	79(16.46)	1.59(1.15–2.21)	0.004*
	CC	172(67.98)	153(63.75)	Ref	
rs1800469	CT	70(27.66)	73(30.41)	0.85(0.56–1.28)	0.48
	TT	11(4.34)	14(5.83)	0.69(0.27–1.71)	0.41
	C	414(81.81)	379(78.95)	Ref	
	T	92(18.19)	101(21.05)	0.83(0.6–1.15)	0.26
	AA	109(43.08)	131(54.58)	Ref	
rs2430561	AT	111(43.87)	96(40)	1.38(0.94–2.05)	0.08
	TT	33(13.04)	13(5.42)	3.05(1.47–6.62)	0.001*
	A	329(65.01)	358(74.58)	Ref	
	T	177(34.99)	122(25.42)	1.58(1.88–2.09)	0.001*

*Significant p value after Bonferroni correction (i.e., the adjusted p value at the 0.05 significance level is 0.016. Allele C (rs833061), T (rs1800469), and T (rs2430561 are responsible for increased production of these cytokines and are considered as risk allele. Hence, the TT, CC and AA genotypes and T, C, and A alleles of rs833061, rs1800469 and rs2430561, respectively, are considered as reference genotype and allele.

No significant association was observed in case of rs1800469 C/T among the PDR and DC groups. Multiple testing adjustment was done using the Bonferroni method.

The rs833061 CC (PDR: 7.92% versus DC: 2.5%; p=0.0042) genotype along with the C allele (PDR: 23.92% versus DC: 16.46%; p=0.0043) was significantly associated with PDR.

The rs2430561 TT genotype was significantly overrepresented in patients with PDR (PDR: 13.04% versus DC: 5.42%; p=0.0012) when compared with the diabetic controls. T allele frequency was significantly higher between the PDR group compared to the controls (PDR: 34.99% versus DC: 25.42%; p=0.0011).

### Association analysis of rs833061 T/C and rs2430561 A/T using logistic regression

The logistic regression analysis assuming a dominant mode of inheritance revealed that there was significant association of the two SNPs with PDR occurrence (p=0.02, OR=2.32 for rs833061 C and p=0.0001, OR=4.11 for rs2430561 T), but the duration of DM had no significant effect (p=0.44) on PDR occurrence (Table 3). The analysis reveals that the two SNPs had no significant interaction effect (p=0.72) present on PDR occurrence (as given in Table 4).

**Table 3:** Association of rs833061T/C and rs2430561A/T.

Independent variables	Regression coefficient	Z value	Odds ratio (95% confidence interval)	P value
Duration of diabetes	0.013	0.77	1.01 (0.9794 – 1.0485)	0.44
rs833061	0.48	2.32	1.62 (1.0777 – 2.4277)	0.02*
rs2430561	0.93	4.11	2.54 (1.6296 – 3.9669)	0.0001*

*p value significant at 0.05 level. Logistic regression analysis was performed on case (PDR=253 subjects) control (DC=240) data as the dependent variable and VEGF – 460T/C rs833061) and IFNγ + 874A/ T (rs2430561) genotypes along with duration of disease as independent variable to study them as predictors for the development of PDR in type 2 diabetic subjects. Dominant model was used for the SNPs.

**Table 4:** Interaction of the two associated SNPs.

Independent variables	Regression coefficient	t value	P value
Interaction of rs833061/rs2430561	0.018	0.35	0.73

Linear regression analysis was performed on the residuals by taking the interaction effects of the three SNPs [VEGF – 460T/C (rs833061), and IFNγ + 874 A/T (rs2430561)] as the independent variables after regressing out the main effect of three SNPs.

### Genetics of early development of proliferative diabetic retinopathy

Genotypic and Allelic frequency distribution of rs833061 and rs2430561 were compared among two discordant groups, where groups are defined as Diabetic subjects who develop PDR within 10 year duration of established type2 DM (n=60) and the diabetic controls (DC) who did not develop this complication after 20 years or more (n=68; Table 5). The VEGF rs833061 TT genotype and the rs833061 CC genotype frequencies were significantly higher among the early patients with PDR compared with the late DM controls (21.67% versus 5.88%; p=0.005). The rs833061 C allele frequency was also significantly higher among the early subjects with PDR (40% versus 20.59%; p=0.0009) compared to the late DM controls. The rs2430561T allele frequency was significantly over represented among PDR patients [Cases: 35% versus Controls: 19.12%; p=0.0046]. The study also demonstrated that the IFN-γ +874TT genotype frequency [Cases: 16.67% versus Controls: 4.42%; p=0.0144] significantly increased among the PDR patients.

**Table 5:** Genotypic and Allelic frequency distribution among two discordant groups.

dBSNP	Genotype	PDR n=60(%)	DC n=68(%)	OR(95%CI)	P
rs833061	TT	25(41.67)	44(64.71)	Ref	
	CT	22(36.66)	20(29.41)	1.93 (0.82–4.54)	0.11
	CC	13(21.67)	4(5.88)	5.72(1.15–26.16)	0.005*
	T	72(60)	108(79.41)	Ref	
	C	48(40)	28(20.59)	2.57(1.42–4.65)	0.0009*
rs2430561	CC	28(46.66)	45(66.17)	Ref	
	CT	22(36.67)	20(29.41)	1.76(0.76–4.08)	0.17
	TT	10(16.67)	3(4.42)	5.35(1.21–32.24)	0.014*
	C	78(65)	110(80.88)	Ref	
	T	42(35)	26(19.12)	2.28(1.24–4.2)	0.004*

*Significant p value after Bonferroni correction (i.e., the adjusted p value at the 0.05 significance level is 0.016). Genotypic and Allelic frequency distribution of rs833061 and rs2430561 among two discordant groups, where groups are defined as Diabetic subjects who develop PDR within 10 year duration of established type2 DM and the diabetic controls (DC) who did not develop this complication after 20 years or more.

Interestingly it is observed that the odds ratio of rs833061CC genotype was increased by almost twofolds in PDR subjects (developed PDR at10 years or below 10 years) compared with the total study group. This data reveals that the rs833061CC genotype increased the risk of early development of PDR.

Table 6 shows the distribution of the low expressive genotype, i.e., rs833061 TT and rs2430561 AA among patients with early PDR (developed PDR at 10 years or less) and diabetic control patients (did not develop retinopathy after 20 years or longer duration of DM). The low expressive genotype, i.e., the rs833061TT genotype, was significantly higher among the DC group compared to the PDR group (PDR: 41.67% versus DC: 64.71%; p=0.0126 for rs833061 TT, PDR: 46.66% versus DC: 66.17%; p=0.03 for rs2430561 AA). This result reflects the phenomenon that rs833061 TT (the low expressive genotype) may provide protection against development of PDR in patients with longstanding type 2 diabetes (duration of DM 20 years or more). All p values reported in Table 2, Table 5, and Table 6 (rs833061, rs1800469, rs361525, and rs2430561) are significant at the 5% level even after Bonferroni correction for multiple testing.

**Table 6:** Distribution of low expressive genotype between discordant groups.

dB SNP	PDR n=60 (%)	DC n=68 (%)	OR (95% Confidence interval)	P value
rs833061TT	25 (41.67)	44 (64.71)	0.39(0.19 – 0.796)	0.012*
rs2430561AA	28 (46.66)	45 (66.17)	0.44(0.22 – 0.91)	0.032

*Significant p value after Bonferroni’s correction (i.e the adjusted p value at the 0.025 significance level is 0.016).

### Combined genotype analysis

The frequency distribution of the combined genotype groups (as discussed in detail in the Methods section) among the cases and controls is given in Table 7. The logistic regression analysis revealed that the categorical variable, which we defined as the number of loci where an individual carries the homozygous genotype of the putative disease-causing allele, is significantly associated with the case status (p=5.91×10^−6^). As the number of dominant allele increases by a unit, the log odds of being affected increases by 0.7545 (Table 8). The number of dominant alleles in the combination genotype also decreases among the controls when compared with the cases.

**Table 7:** Frequency distribution of “Combination” genotype.

Category using combination genotype (combination)	PDR n=253(%)	DC n=240(%)
1 (all the three SNPs are dominant)	5(1.98)	1(0.42)
2 (two SNPs has dominant genotypes)	135(53.36)	66(27.5)
3 (one of the SNP has dominant genotype)	83(32.81)	117(48.75)
4 (none of the SNP has dominant genotype)	30(11.85)	56(23.33)

Combined genotype analysis with cases and controls were done by assuming dominant model of inheritance. Categorical variable were done with four categories according to the number of dominant genotypes present in the combination genotype (3 if all the three SNP have dominant genotype; 2 if two SNPs have dominant genotypes; 1 if only one of the SNPs has dominant genotype; 0 if none of the SNPs has dominant genotype).

**Table 8:** Association of combined genotypes.

Variable	Estimate	Standard error of beta	P value
Intercept	−0.896	0.19	2.02×10^−6^
combination	0.754	0.13	5.91×10^−9^

Logistic regression with “logit” link function: Using the variable combination, logistic regression model was fit by taking the above mentioned variable as the predictor and regressing on the case control status.

## Discussion

In the past three decades, many growth factors and cytokines, most of which have been implicated in having a role in the development of PDR, have been discovered. Multiple interactive mechanisms may come into play leading to cellular damage and adaptive changes leading to the development of this devastating complication of diabetes. In this regard, identifying genetic markers, which facilitate the risk of developing disease particularly for multifactorial diseases such PDR, is essential for treatment and prevention.

The concept of growth factor-mediated retinal angiogenesis was postulated by Michaelson [15]. Recently, attention has focused on VEGF as the most potent modulator of intraocular angiogenesis and permeability in PDR. Several features of VEGF make it a plausible mediator of retinal neovascularization and vascular permeability in the ischemic ocular condition. VEGF is produced from several ocular cells along with the expression of abundant VEGF receptors by the retinal endothelial cells [16,17]. In response to prolonged hypoxia, VEGF is markedly upregulated [7]; however, hypoxia may not be the only factor for upregulation and expression of VEGF. It is further controlled by its promoter polymorphisms, as the *VEGF* gene is unusually polymorphic in the promoter region. Several studies have focused on SNPs within the promoter region and their association with angiogenic eye disease. Previously, strong association has been reported at the VEGF −634G/C locus among patients with PDR in a Brazilian cohort of patients of European ancestry [18] with types 2 diabetes. The data suggest that the VEGF −634C allele increases the chance of an individual of developing PDR by 1.9 times [18,19]. Churchill et al. demonstrated that carrying the VEGF-152A (rs13207351) and −116A (rs1570360) alleles was significantly associated with PDR [20]. Among several polymorphic loci, the transcription start site at rs833061 of this gene representing a transition from nucleotide thymine to cytosine affects the transcription of VEGF, and rs833061 C showed 70% increased promoter activity over rs833061 T [21]. Our study indicates that the homozygous rs833061 CC genotype along with C allele distribution is significantly increased among patients with PDR. In addition to prolonged hyperglycemia, the influence of the rs833061 C allele may play an important role in accelerating the pathogenesis of PDR via increased activation of VEGF. Interestingly, our study replicates the finding among UK Caucasian individuals with type 2 diabetes who develop this retinal complication [22].

In addition to hypoxia and genetic control, VEGF has been shown to be upregulated by several growth factors and cytokines. In particular, TGF-β1 activates the expression of VEGF most effectively in human retinal pigment epithelial cells [8]. TGF-β1 itself also acts as an important agent for modulating ocular cell migration and proliferation by inducing growth factor-like fibroblast growth factor and platelet-derived growth factor, all of which may accelerate the process of retinal neovascularization [23]. TGF-β1 is again involved in deposition of extracellular matrix (an essential step in new vessel formation) and can play a crucial role by stimulating angiogenesis and inhibiting the endothelial function of the eye in patients with retinal ischemia and patients with PDR [24]. Active TGF-β1 concentration in plasma and cells partially depends on the TGF-β1 promoter polymorphism at the rs1800469 locus where the homozygous TT genotype shows increased concentration of active TGF-β1 (two times its homozygous CC genotype) [25]. Therefore, predisposition to various forms of disease related to angiogenesis may be correlated with the presence of the particular alleles at the rs1800469 locus as was found in atherosclerosis [25,26]. In the present study, we focused on this locus to find a possible association with intraocular angiogenesis of patients with type 2 diabetes but did not find a significant association at either the genotype or allelic level with PDR. Early development of PDR is associated with increased production of several inflammatory mediators. During the last few years, cytokines such as TNF-α and IFN-γ have been shown to be involved in pathogenesis of PDR [27]. TNF-α, the proinflammatory cytokine, acts as an important immunomodulator in playing a potent role in retinal neovascularization and fibroplasias [28]. Our previous study demonstrated that allele A at rs361525 (TNF-α-238G/A), which was also previously reported as a high expression variant [29], was strongly associated with PDR, at the genotype level (AA) and the allelic level (A allele) [30]. However, we did not find a significant association with the alleles of rs1800629 (TNF-α-308G/A) and the disease [30]. In contrast, a previous functional study demonstrated that the rs2430561T allele (binding site for nuclear factor-κB) influences higher production of IFN-γ [31]. Increased production of IFN-γ may also influence the activation of TNF-α through maintaining its mRNA stability [32]. It has been generally believed that angiogenesis is the result of vigorously maintained equilibrium between activities of positive and negative regulators such as TNF-α, interleukin 8 (IL-8), and VEGF (positive regulators) versus negative regulators such as pigment epithelium derived growth factor (PEDF) and interferon-inducible protein 10 (IP-10) [33]. Proinflammatory cytokines such as IL-12, IFN-γ, IP-10, and IL-18 appear to be related to the angiogenic program [33]. To the best of our knowledge, the present study is the first report of a polymorphism in the intron of the IFN-γ gene, which is shown to be strongly associated with PDR. Hence, we may interpret that rs833061 T may be involved in activating IFN-γ to increase the risk for the pathogenesis of PDR through the process of inflammation and VEGF activation.

From logistic regression analysis, the significant increased association of the high expressive genotype of the two SNPs (rs833061 and rs2430561) with PDR occurrence might reflect the phenomenon that increased production of these cytokines may play a crucial role in the genesis of retinal neovascularization directly or through the activation of VEGF. A previous study demonstrated that upregulation of TNF-α and IFN-γ induces VEGF mRNA expression directly or through TGF-β1 upregulation [8]. However, the present study did not reveal a significant association of the interaction effect of these two SNPs and PDR occurrence. The combined genotype association of the SNPs revealed that the number of dominant alleles in the combination genotype significantly decreased among the DM group (controls) when compared with the PDR group (cases). This supports the dominant mode of inheritance we assumed for all three SNPs. More importantly, it emphasizes that the SNPs may be part of a complex pathway. Though each SNP confers a slight increase in risk for PDR, when they are combined, the chances of developing PDR increase considerably.

The risk of developing PDR increases with poor glycemic control over a longer period of time [34]. However, in the present study some diabetic individuals (n=60) developed this retinal complication within 10 years duration of DM while a group of diabetic individuals (n=68) protected themselves from DR 20 years after inception of type 2 DM despite having similar glycemic control. It might be due to the complex interplay of environmental and genetic factors for the disease pathology. To get the proper signal or genetic effect for early development of PDR, we compared the allelic distribution of rs833061 and rs2430561 between the patients with PDR who developed this complication within a short period (n=60; 10 years or less) and the control patients, who did not develop this complication for longer duration of DM (20 years or more; n=68) with matched glycemic and nutritional status (Table 5 and Table 6).

The study revealed that the risk of developing PDR on early onset of diabetes (within 10 years of inception of type 2 DM) due to the rs833061 CC and rs2430561 TT genotypes increased 5.72- and 5.35-fold, respectively, compared to diabetic individuals who did not develop this complication within 20 years or more. The study also reflects the phenomenon that rs833061 TT (low expressive genotype) may provide protection against developing PDR for patients with longstanding type 2 diabetes (duration of diabetes 20 years or more) compared to diabetic individuals who developed PDR within 10 years and is independent of glycemic control.

A previous study demonstrated that prolonged hyperglycemia is an important risk factor for the pathogenesis of PDR [34]. However, hyperglycemia is not the only factor for developing this microvascular complication. The study demonstrated that the genetic variations namely the rs833061 C and rs2430561 T alleles are the potent risk factors for the pathogenesis of PDR. Our present study might be used as a tool in predicting, preventing, and managing PDR and might be useful as target discovery for developing novel therapeutics.
